# Control procedures and estimators of the false discovery rate and their application in low-dimensional settings: an empirical investigation

**DOI:** 10.1186/s12859-018-2081-x

**Published:** 2018-03-02

**Authors:** Regina Brinster, Anna Köttgen, Bamidele O. Tayo, Martin Schumacher, Peggy Sekula, on behalf of the CKDGen Consortium

**Affiliations:** 1 Institute of Medical Biometry and Informatics, University of Heidelberg, Im Neuenheimer Feld 130.3, 69120 Heidelberg, Germany; 2 Institute for Medical Biometry and Statistics, Faculty of Medicine and Medical Center, University of Freiburg, Stefan-Meier-Str. 26, 79104 Freiburg, Germany; 3 Institute of Genetic Epidemiology, Faculty of Medicine and Medical Center, University of Freiburg, Hugstetter Str. 49, 79106 Freiburg, Germany; 4 Department of Public Health Sciences, Loyola University Chicago Stritch School of Medicine, Maywood, IL USA

**Keywords:** False discovery rate, Simulation study, Low-dimensional setting, Q-value method

## Abstract

**Background:**

When many (up to millions) of statistical tests are conducted in discovery set analyses such as genome-wide association studies (GWAS), approaches controlling family-wise error rate (FWER) or false discovery rate (FDR) are required to reduce the number of false positive decisions. Some methods were specifically developed in the context of high-dimensional settings and partially rely on the estimation of the proportion of true null hypotheses. However, these approaches are also applied in low-dimensional settings such as replication set analyses that might be restricted to a small number of specific hypotheses. The aim of this study was to compare different approaches in low-dimensional settings using (a) real data from the CKDGen Consortium and (b) a simulation study.

**Results:**

In both application and simulation FWER approaches were less powerful compared to FDR control methods, whether a larger number of hypotheses were tested or not. Most powerful was the q-value method. However, the specificity of this method to maintain true null hypotheses was especially decreased when the number of tested hypotheses was small. In this low-dimensional situation, estimation of the proportion of true null hypotheses was biased.

**Conclusions:**

The results highlight the importance of a sizeable data set for a reliable estimation of the proportion of true null hypotheses. Consequently, methods relying on this estimation should only be applied in high-dimensional settings. Furthermore, if the focus lies on testing of a small number of hypotheses such as in replication settings, FWER methods rather than FDR methods should be preferred to maintain high specificity.

**Electronic supplementary material:**

The online version of this article (10.1186/s12859-018-2081-x) contains supplementary material, which is available to authorized users.

## Background

Advances in molecular biology and laboratory techniques allow for evaluating a multitude of different features in humans on a large scale to elucidate (patho-)physiology and risk factors for a specific disease or its progression. In recent studies, up to millions of features are often assessed simultaneously in discovery set analyses such as in genome-wide association studies (GWAS) where single nucleotide polymorphisms (SNPs) are evaluated with respect to a single trait or clinical outcome [1]. For reasons of practicability, the usual analysis procedure of such high-dimensional data comprises statistical testing of each single feature separately with the outcome of interest [2].

Statistical testing aims to verify a hypothesis, which is either rejected or accepted based on the observed test statistic [3]. Depending on the decision, there are two possible mistakes that can occur: The null hypothesis might be erroneously rejected although it is true (false positive decision, type I error) or failed to reject although it is false (false negative decision, type II error). The type I error can be controlled by defining a significance threshold. For a single hypothesis, a commonly used threshold is α=0.05. However, when testing multiple hypotheses such as in GWAS, the application of a threshold like 0.05 across all tests will result in an unacceptable large number of false positive results. Consequently, other ways to control the type I error are required.

In general, there are different approaches: the control of the family-wise error rate (FWER) and the control or the estimation of the false discovery rate (FDR) [4]. FWER methods such as the well-known Bonferroni correction [5] were already proposed when the number of tested hypotheses was not as large as, for example, in GWAS nowadays. Although often applied, these methods are thought to be too conservative in a high-dimensional setting. Alternatively, FDR methods that are less conservative and partially developed in the context of high-dimensional data can be used. In addition, there are approaches to estimate a significance measure for each individual hypothesis, such as the local false discovery rate (LFDR) [6] and the q-value [7].

FDR methods are also used quite frequently nowadays and not only in high-dimensional settings but also in situations where the number of assessed features is small such as in a replication set analysis restricted to the significant hypotheses of the discovery set analysis. For a small number of features, however, there are limited data on the performance of FDR methods. The aim of this study was thus to assess FDR methods in low-dimensional data and to compare them to classic FWER methods. For this purpose, we used real data obtained from the CKDGen Consortium [8] to illustrate the different control methods. Moreover, we conducted a simulation study to evaluate different control methods in different settings.

## Methods

### Control methods

In order to describe different error control and estimation methods, we adopted the notation of Benjamini and Hochberg [9] on test decisions (Table 1). Assuming *m* hypotheses H_1_, …, H_m_ were tested leading to the observation of the respective *m p*-values p_1_, …, p_m_. If the truth would be known, type I errors are described by *V* and type II errors by *T*. However, only *m* and the total number of rejections, *R*, are observable in practice. The overall significance threshold is called α.

**Table 1:** Statistical hypothesis test with possible test decisions related to the unknown truth (notation)

		Test decision		
		*declared non-significant*	*declared significant*	Total
Underlying truth	*true null*	*U*	*V* (type I error, *α*)	*m*_0_
	*non-null/alternative*	*T* (type II error, *β*)	*S*	*m*_1_
Total		*m-R*	*R*	*m*

#### Methods controlling the family-wise error rate (FWER)

FWER is defined as the probability of making at least one false positive decision: *FWER* = Pr(*V* > 0). The error rate can be controlled by a fixed threshold α. In the following, four well known methods are considered (Table 2a):

**Table 2:**
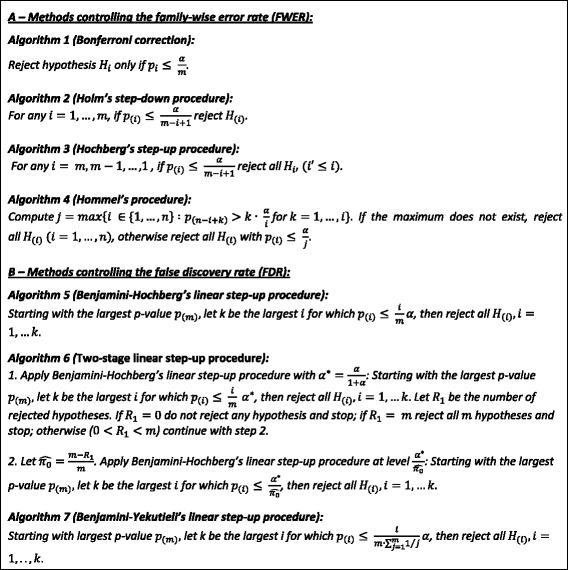
Algorithms of methods controlling family-wise error rate (FWER) and false discovery rate (FDR) Let *m* be the number of hypotheses H_1_, …, H_m_ to test and p_1_, …, p_m_ their respective *m p*-values. The *p*-values ranked in increasing order are defined as p_(1)_ ≤ … ≤ p_(m)_. The overall significance threshold is called α. Furthermore, let $\widehat{\pi_0}$ be the estimated proportion of true null hypotheses

The simplest and likely most often applied control method of the FWER is the ***Bonferroni correction*** [10]. It compares each individual *p*-value p_1_, …, p_m_ with the fixed threshold$\frac{\upalpha}{\mathrm{m}}$. *P*-values that are smaller than the threshold lead to the rejection of the respective null hypothesis. The Bonferroni correction guarantees the control of the FWER at level α in a strong sense, which means that the control is ensured for every proportion of true null hypotheses. Bonferroni correction does not demand independent *p*-values and hence can be applied to any dependency structures. Nevertheless, Bonferroni can be conservative; true alternatives might therefore be missed.

To reduce the number of missed true alternatives, approaches to adjust Bonferroni correction were proposed that use the number of independent tests (also: effective number) instead of the actual number of conducted tests (e.g. Li et al. [11]). Therefore, these approaches gain in power over the traditional Bonferroni correction. In the specific context of GWAS, for example, an adjusted Bonferroni correction frequently applied was proposed by Pe’er et al. [12] that accounts for correlation between SNPs due to linkage disequilibrium (LD) by estimating the number of independent genome-wide loci (*n* = 1,000,000 in individuals of European ancestry). Instead of using the much larger number of all SNPs tested for association (often several millions), the overall significance threshold such as α=0.05 is divided by the number of independent SNPs to define an adjusted significance threshold. For GWAS on Europeans, for example, the significance threshold becomes $\frac{0.05}{\mathrm{1,000,000}}=5\times {10}^{-8}$. Similarly, the number of independent tests in the field of metabolomics can be estimated with help of principle component analysis to reduce the number of all tests used in Bonferroni correction (e.g. Grams et al. [13]).

The other three FWER control methods considered below are sequential methods for which *p*-values need to be ranked in increasing order: p_(1)_ ≤ … ≤ p_(m)_.

***Holm’s step-down procedure*** [10] rejects at least as many hypotheses as Bonferroni correction does. The gain in power of Holm’s procedure by defining more features significant is larger with larger number of alternative hypotheses. Like the Bonferroni correction, Holm’s procedure has no restrictions with respect to the dependency structure of *p*-values.

***Hochberg’s step-up procedure*** [14] and also ***Hommel’s procedure*** [15] make use of the assumption that the p-values under the true null hypotheses hold a positive regression dependency. Positive dependency structure assumes the probability of a p-value belonging to the null hypothesis to be increasing with increasing p-value. In situations of a positive dependency structure, Hochberg’s procedure is more powerful than Holm’s [4]. Hommel’s procedure, however, is the most powerful FWER control procedure of the previously mentioned methods when the assumption holds since it rejects at least as many hypotheses as Hochberg does. One criticism of the method lies in the higher computational load.

#### Methods controlling the false discovery rate (FDR)

In contrast to FWER, the false discovery rate (FDR) represents the proportion of false positives. This error rate is defined as following: $FDR=E\left[\frac{V}{R}|R>0\right]\Pr \left(R>0\right).$ FDR can be controlled at a fixed significance threshold as well. Furthermore, Benjamini and Hochberg [9] proved that every FWER control method controls the FDR likewise. The three most common FDR control methods that also require ordered *p*-values are considered below (Table 2b):

***Benjamini-Hochberg’s linear step-up procedure*** [9] controls the FDR at level α assuming positive dependent p-values (see description above) under the true null hypotheses such as Hommel’s and Hochberg’s FWER procedures. It shows greater power than any of the above mentioned FWER methods.

The ***two-stage linear step-up procedure*** [16] is an adapted procedure of Benjamini-Hochberg’s that takes the estimation of the proportion of the true null hypotheses, *π*_0_, into account. The gain in power of the two-stage procedure compared to the classical Benjamini-Hochberg’s linear step-up procedure is dependent on the proportion of true null hypotheses (*π*_0_) [4]. For *π*_0_ close to 1, the adapted version has low power. The adaptive approach has been proven for independent *p*-values only.

Finally, ***Benjamini-Yekutieli’s linear step-up procedure*** [17] has no restrictions on the dependency structure of p-values at all. It is more conservative compared to the Benjamini-Hochberg’s linear step-up procedure [4] and the two-stage linear step-up procedure [16].

#### Methods estimating the false discovery rate (FDR)

Recent approaches do not control the FDR in the traditional sense, but rather estimate the proportion of false discoveries. In order to estimate the FDR, the estimation of the proportion of the true null hypotheses, *π*_0_, is conducted first which can lead to a gain in power compared to the classic FWER and FDR control methods. Two common FDR estimation methods are described in the following:

***Storey’s q-value method*** [7] uses a Bayesian approach to estimate the so-called positive false discovery rate (pFDR), a modified definition of the false discovery rate which assumes at least one rejection: $pFDR=E\left[\frac{V}{R}|R>0\right]$. The approach is based on the idea of estimating the pFDR for a particular rejection region, *γ*, to achieve a control of the pFDR. In order to determine a rejection region, the q-value was introduced as the pFDR analogue of the *p*-value. The q-value provides an error measure for each observed p-value. It denotes the smallest pFDR that can occur when calling that particular p-value significant: $q(p)=\underset{\left\{\gamma \ge p\right\}}{\min } pFDR\left(\gamma \right)$. The approach assumes independent, respectively “weak dependent” *p*-values, whose dependency effect becomes negligible for a large number of *p*-values [18]. The method provides an improvement in power compared to the classic Benjamini-Hochberg’s linear step-up procedure due to its estimation of *π*_0_ [7].

Likewise, Strimmer [19] proposed an alternative method to estimate q-values based on pFDR (***Strimmer’s q-value method***). In addition, the method provides estimates of the so-called local false discovery rate (LFDR, ***Strimmer’s LFDR approach***) that again present individual significance measures such as the q-values for each *p*-value. It describes the probability that a p-value leads to a false positive decision given the observed data information. Estimations are based on a Bayesian approach using a modified Grenander density estimator [19].

#### Software implementation

R packages are available for all described control methods via CRAN [20] or Bioconductor [21]. Specifically, we used the packages *multtest* [22]*, qvalue* [23] (Bioconductor), *mutoss* [24] and *fdrtool* [25] (CRAN) in our study. We applied the methods using default options of the packages. However, Storey’s q-value application displayed an error whenever the estimated proportion of true null hypotheses (*π*_0_) was close to zero, which occurred when all *p*-values happened to be (very) small. Therefore, we adjusted the range of input p-values (“lambda”) in a stepwise manner until the application allowed the estimation of *π*_0_. Further details on our R-code and the stepwise algorithm can be obtained directly from the authors. Statistical significance using either FWER, FDR controlling or FDR estimation methods such as the q-value methods or LFDR, was defined as a cutoff of 0.05.

### Data example

For illustration of the different control methods, we obtained data from the CKDGen Consortium [8]. The aim of this project was to identify genetic variants associated with estimated glomerular filtration rate (eGFR), a measure for kidney function, and chronic kidney disease (CKD). Altogether, 48 study groups provided genome-wide summary statistics (GWAS results) from 50 study populations for SNP associations with eGFR based on serum creatinine (eGFRcrea) (2 study groups provided GWAS results for 2 subpopulations separately). The discovery meta-analysis of all GWAS was carried out using an inverse variance-weighted fixed effect model and incorporated data from 133,413 individuals of European ancestry. Genomic control had been applied before and also after meta-analysis to reduce inflation and thus limit the possibility of false positive results. In the meta-analysis, 29 previously identified loci and 34 independent novel loci (*p*-value < 10^−6^) were detected. Novel loci were then verified in an independent replication set (14 studies; *N* = 42,166). For 16 of the 34 novel loci, replication analysis showed direction-consistent results with p-value combining discovery and replication < 5×10^−8^ (see Table 1 in Pattaro et al. [8]). For all but 1 SNP (rs6795744), the reported q-values in the replication study were < 0.05.

The results of the discovery meta-analyses for different traits including eGFRcrea (approximately 2.2 million SNPs) are publicly available [26]. Moreover, we obtained the summary statistics from GWAS results for eGFRcrea of all studies contributing to the discovery (48 studies, 50 result files) for our project. For the illustration of the different control methods in both discovery (high-dimensional) setting and replication (low-dimensional) setting, we split the 50 study contributions into two sets taking into account general study characteristics (population-based study versus diseased cohort) and imputation reference (HapMap versus 1000 Genomes [27]). By conditioning on the presence of at least one study from each of the 4 categories in either setting and on a sample size ratio of 2:1, study contributions were randomly assigned to discovery set or replication set. The final discovery set contained 35 studies with 90,565 individuals (67.9%) and the replication set 15 studies with 42,848 individuals (32.1%).

Based on the same set of SNPs as in the publicly available data set, our discovery set was processed similarly to the original analysis [8] by using an inverse variance-weighted fixed effect model and genomic control before and after that step. For simplicity reasons we considered two-sided *p*-values in the discovery and replication set analysis. To select independently associated SNPs, SNPs were clustered based on LD pruning using the *--clump* command of Plink v1.90b2 (r^2^: 0.2, window: 1000 kb, significance threshold for index SNP: 10^−6^) [28] and data of 1000 Genomes project (phase 3) as the LD reference. SNPs with the lowest *p*-value within a specific region were considered as index SNPs. Few SNPs that were either not present in the reference or tri-allelic were excluded at this point. Using the prepared discovery data, the various FDR and FWER methods were then applied exploratively.

Similar to the published analysis by the CKDGen Consortium (Pattaro et al. [8]), independent index SNPs with *p*-value < 10^−6^ were selected from the discovery set to be followed up in the replication set. The various control methods were subsequently applied to the results of the meta-analysis (same model as before but without genomic control) in the replication set to identify significant findings.

### Simulation study

In order to assess power and specificity of the described FWER and FDR methods in detail, we conducted a simulation study with varying settings, with special emphasis on situations with a smaller number of tested features. The R-code of the simulation study can be requested from the author.

For this purpose, test statistics for varying numbers of features (*N* = 4, 8, 16, 32, 64, 1000) were simulated to generate data sets. Test statistics for single features were simulated by drawing from $\mathcal{N}\left(\upbeta, 1\right)$ with either *β* = 0 (null hypothesis) or β ∈ {1.0, 2.5} (alternative or non-null hypothesis). Depending on the number of features in a given data set, the proportion of the true null hypotheses π_0_ ∈ {25%, 50%, 75%, 100%} was a-priori defined. Each scenario defined by the different combinations of parameters was repeated 100 times. In preparation of the subsequent application of control methods, simulated test statistics were transformed into two-sided *p*-values.

The power of each approach was defined as proportion of correctly rejected hypotheses among all true alternative hypotheses whereas the specificity was defined as the proportion of correctly maintained hypotheses among all true null hypotheses. Furthermore, we evaluated the estimation results of the proportion of true null hypotheses of Storey’s and Strimmer’s q-value methods within the simulation study.

## Results

### Data example

For the purpose of illustration, the 50 GWAS summary statistics provided by contributing study groups included in the original CKDGen discovery meta-analysis of eGFRcrea were split into 2 sets resembling a high-dimensional discovery set (35 studies, 90,565 individuals) and a low-dimensional replication set (15 studies, 42,848 individuals). Details on the two sets are provided in Additional file 1 and Additional file 2.

Similar to the published analysis by the CKDGen Consortium (Pattaro et al. [8]), the discovery set was processed to select independent variants to be moved forward to a low-dimensional replication analysis. Based on *p*-value threshold < 10^−6^ followed by LD pruning, 57 index SNPs from different genomic regions were selected from the discovery set. The replication analysis of the 57 selected index SNPs showed direction-consistent effect estimates for 56 SNPs.

**Fig. 1:**
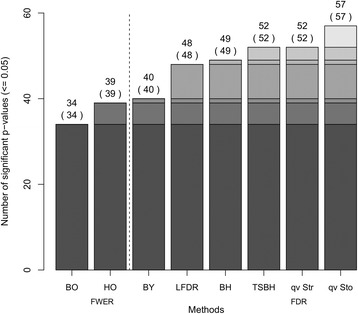
CKDGen data example – Number of significant *p*-values (regions) in replication set. Applied procedures controlling the type I error: Bonferroni correction (BO), Hommel’s procedure (HO), Benjamini-Yekutieli’s procedure (BY), Strimmer’s LFDR method (LFDR), Benjamini-Hochberg’s procedure (BH), Two-stage procedure (TSBH), Strimmer’s q-value method (qv Str), Storey’s q-value method (qv Sto). Results are ordered by number of significant p-values leading to a separation of FDR methods from FWER methods (indicated by dashed line). Additional significant p-values from one approach to another are indicated by decreasing gray shades within the bars

Subsequently, the various control methods were applied to the meta-analysis results of the replication set to identify significant findings. Figure 1 presents the number of significant results of the different control procedures. Since the FWER methods Holm, Hochberg, and Hommel declared the same *p*-values as significant, we decided to display the performance of Hommel’s approach only.

In contrast to FDR methods, FWER methods rejected the smallest number of hypotheses with Bonferroni being least powerful. Among the FDR methods, FDR estimating methods by Strimmer and Storey provided more power. Storey’s q-value method rejected all hypotheses and it was the only approach which declared the direction-inconsistent SNP as significant.

As expected, the applied FWER and FDR methods showed a monotone subset behavior related to rejected hypotheses, i.e. that the *p*-values declared significant from a more conservative approach were always included in the set of p-values declared significant from a less conservative method. This is a consequence of the methods’ property that – if a specific p-value is declared significant – all other smaller p-values are also declared significant.

### Simulation study

#### Power and specificity of control methods

**Fig. 2:**
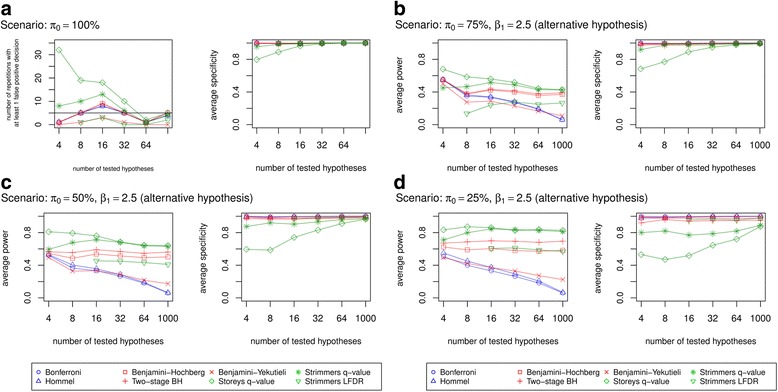
Simulation – Number of repetitions with at least 1 false positive decision and average specificity for π_0_ = 100% (**a**). Average power and specificity for β_1_ = 2.5 and π_0_ = 75% (**b**), 50% (**c**), 25% (**d**). Applied procedures controlling the type I error: Bonferroni correction, Hommel’s procedure, Benjamini-Hochberg’s procedure, Two-stage procedure, Benjamini-Yekutieli’s procedure, Storey’s q-value method, Strimmer’s q-value method, Strimmer’s LFDR method. Power is defined as the proportion of correctly rejected hypotheses and specificity as the proportion of correctly maintained hypotheses. Both proportions potentially range from 0 to 1. Simulations for each scenario were repeated 100 times

In a setting where the proportion of true null hypotheses, π_0_, is 100%, Storey’s and Strimmer’s q-value methods most often falsely rejected true null hypotheses when the number of tested hypotheses *N* is small (≤32), while for larger numbers of tested hypotheses and/or other methods the number of erroneous decisions mostly did not exceed 5 (Fig. 2a). Benjamini-Yekutieli’s procedure and Strimmer’s LFDR approach performed best with 0 to 3 repetitions of falsely rejected hypotheses for all *N*. As a remark, Strimmer’s LFDR approach could not provide any results for *N* = 4. Specificity of methods to correctly maintain hypotheses is similarly good on average; only Storey’s q-value method showed decreased specificity when the number of tested hypotheses was small.

When the proportion of true null hypotheses was < 100%, the power to correctly reject hypotheses was dependent on π_0_, the effect size (β) and *N*. On average, it increased with decreasing π_0_, increasing β and decreasing *N* overall. Figure 2b, c and d exemplarily show the average power for varying π_0_ and *β*_1_ = 2.5 under the alternative hypothesis, in dependence on *N*. Further figures for an effect size of *β*_1_= 1 can be found in the Additional file 3.

As expected, FDR methods, especially the two q-values methods, were more powerful than FWER methods. In terms of specificity, Storey’s q-value method followed by Strimmer’s q-value method showed lower specificity results for small *N* (≤16) than other methods. We observed similarity in specificities among the other methods. Again, Strimmer’s LFDR approach did not provide results when number of hypotheses were < 8 (Fig. 2b) or < 16 (Fig. 2c and d).

#### Estimation of proportion of true null hypotheses

**Fig. 3:**
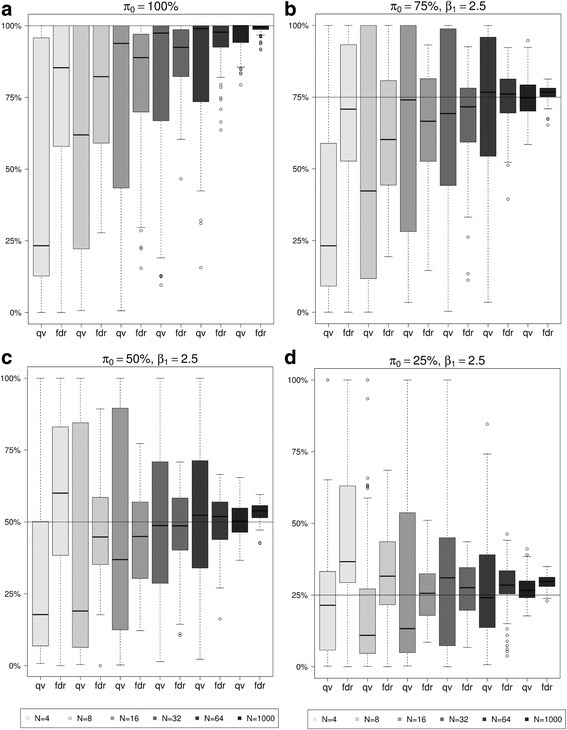
Simulation – Observed estimations of π_0_ for Storey’s (qv) and Strimmer’s q-value methods (fdr) for π_0_ = 100% (**a**) and for β_1_ = 2.5 and π_0_ = 75% (**b**), 50% (**c**), 25% (**d**)

LFDR and q-value methods rely on the estimation of *π*_0_. Figure 3 displays its estimations using Storey’s and Strimmer’s q-value approaches for varying π_0_ and *β*_1_ = 2.5 under the alternative hypotheses (if present), while remaining figures are in the Additional file 4.

For small *N*, both estimations showed large variability within repetitions. Throughout all scenarios, Storey’s method showed greater estimation ranges of *π*_0_ compared to Strimmer’s q-value approach. Moreover, estimation of *π*_0_ was often biased. Only when *β*_1_ = 2.5 and *N* was larger than 32, bias essentially disappeared. When *β*_1_ = 1, however, *π*_0_ was overestimated on average, even for larger *N*.

## Discussion

FDR estimation methods such as Strimmer’s LFDR or Storey’s q-value method have been mainly developed for high-dimensional settings, of which discovery GWAS is one. They provide a less conservative approach compared to standard FWER and FDR control methods. The LFDR as well as the q-value methods are Bayesian approaches which take the whole information on the data itself into account when estimating the proportion of true null hypotheses, *π*_0_. Consequently, for the purposes of FDR estimation, a high-dimensional setting is a great advantage allowing reasonable estimation of *π*_0_. Though controversial, the q-value methods as well as other FDR methods have been used in low-dimensional settings as well, such as in the analysis of replication data sets consisting of only limited number of SNPs. We thus aimed to compare various FWER and FDR methods including the q-value method in order to assess their power and specificity in low-dimensional settings using simulated data and application to real data.

The analysis of our example data from the CKDGen Consortium [8] showed that the FDR estimation methods by Strimmer and Storey declared the largest number of SNPs significant in the low-dimensional replication analysis of 57 SNPs, followed by the FDR control methods of Benjamini-Hochberg and Benjamini-Yekutieli. As expected, the FWER control methods showed the lowest power by declaring the least number of *p*-values significant. Of note, Storey’s q-value method was the only approach which declared the single SNP (rs10201691) that showed direction-inconsistent results between the discovery and replication analyses as significant in the replication analysis.

To deepen the understanding, we conducted a simulation study to systematically assess different scenarios. As one result, the differences between the methods that were seen in the application could be confirmed. For example, Storey’s q-value method showed the highest power especially for a small number of hypotheses. At the same time, however, the specificity results for Storey’s method were lowest when number of tested hypotheses was small. In the presence of alternative hypotheses (*π*_0_ < 100%), we also observed that the FDR methods, Benjamini-Hochberg and the two-stage approach, − although less powerful than both q-value methods – were more powerful than the FWER control methods of Bonferroni and Hommel, but of similar specificity.

Since both q-value methods as well as LFDR rely on the estimation of *π*_0_, we also investigated its estimation accuracy using the different approaches. For both methods, the estimate of *π*_0_ was often biased, especially when numbers of tested hypotheses were small. In addition, Storey’s q-value method showed much higher variance compared to Strimmer’s approach. In summary, the q-value methods rejected in general the largest number of hypotheses which is especially of advantage if researchers wish to obtain a greater pool of significant features to be followed-up in subsequent studies, at the expense of specificity. However, their application should be restricted to high-dimensional settings.

The gain in power for both q-value methods, however, was not observed for LFDR in the simulation study. Strimmer reported the gain in power of the q-value method compared to the LFDR as well and explained it as the tendency of q-values being smaller or equal compared to LFDR for a given set of *p*-values [19]. In the context of gene expression, Lai [29] mentioned a tendency of the q-value to underestimate the true FDR leading to a larger number of low q-values especially when the proportion of differentially expressed genes is small or the overall differential expression signal is weak. We also observed an underestimation in our simulation study, especially for a smaller number of *p*-values. To overcome this issue, Lai [29] suggested a conservative adjustment of the estimation of the proportion of true null hypotheses, the p-values or the number of identified genes.

Moreover, when applying q-value methods or LFDR, correct interpretation of these estimates is requested that is different for the q-values and for LFDR. Strimmer [19] highlighted the easier interpretation of the LFDR compared to the q-value since the LFDR provides point estimates for the proportion of false discoveries for individual hypotheses whereas the q-value of a p-value is the expected proportion of false positives when calling that feature significant [18]. In any case, when applying FDR estimation methods, there is a critical need for a sizeable data set [18, 19]. Storey and Tibshirani [18] described their q-value method as a more explorative tool compared to FWER methods and therefore as well-performing procedure in high-dimensional data. A more recent FDR estimation approach by Stephens [30] provides an alternative to the LFDR, the so called local false sign rate. This empirical Bayes approach describes the probability of making an error in the sign of a certain variant if forced to declare it either as true or false discovery. Simulation studies showed smaller and more accurate estimation of *π*_0_ by Stephens’ approach compared to Storey’s q-value method leading to more significant discoveries [30]. However, small sample sizes represent a challenge for this FDR estimation approach as well.

Another observation of our simulation study worth mentioning was that the FDR method by Benjamini-Yekutieli for arbitrary dependencies, and thus assumed to be more conservative than the Benjamini-Hochberg method, was not only outperformed by this method in terms of power in our application data and simulation, but also less powerful than FWER control methods in some scenarios of our simulation. The latter had already been observed, especially if the expected number of alternative hypotheses is very small [4]. Since Benjamini-Hochberg’s approach controls the FDR at level *π*_0_*α*, adaptive FDR control methods such as the two-stage approach were developed to control the FDR directly at level *α* by taking estimated *π*_0_ into account and thereby gaining power. Especially if *π*_0_ is substantially smaller than 1, the adaptive approaches might outperform Benjamini-Hochberg’s procedure [4].

Before concluding the discussion on results, some limitations of this study warrant mentioning: Although it was important for us to illustrate the effect of the different control methods on the results in real data, observed differences may not be transferrable to every other study setting in general. To overcome this limitation, we conducted a simulation study. Still, the simulation study has limitations of its own: We used a simplified approach to generate data by simulating test statistics rather than analytical data sets to which control methods would have been applied after analysis. Furthermore, we explored a limited set of scenarios and did not consider dependency structures but evaluated *p*-values that were derived from independently simulated test statistics. Hence, additional work could add to the current understanding.

In the face of all the different control methods, it is clear that the decision on what method is actually applied in a given setting should be made not only before the analysis is conducted but also on reasonable ground. Among others, aspects to consider include: (a) the amount of tests to be conducted, (b) the general aim of testing, (c) what is known or can be assumed about dependency structure of *p*-values under the true null hypothesis and (d) what is the assumed proportion of null hypotheses.

If the general aim of the analysis lies on the specific testing of individual hypotheses, FWER control methods should be preferred to FDR control or estimation methods because they provide higher specificity by correctly maintaining true null hypotheses. Within FWER control methods, the power might differ slightly and is, especially, in dependence of given p-value structure. If a positive structure can be assumed, Hochberg’s or Hommel’s procedures are preferable to gain power. The computational burden that comes along with Hommel’s procedure should not be a true issue nowadays. Goeman and Solari [4] especially expected a gain in power of Hochberg’s and Hommel’s compared to Bonferroni’s and Holm’s methods if the proportion of alternative hypotheses is rather large. We, however, observed only a rather small gain in power in our simulation study that might be induced by the simulation of independent test statistics.

If researchers, however, wish to identify a promising set of hypotheses for follow-up rather than specific testing of single hypotheses with high specificity, we agree with Goeman and Solari [4] who recommended the use of FDR control methods. To reach highest power, one may even apply the FDR estimating method of q-values, when the number of tests is reasonably large.

## Conclusions

In summary, our findings highlight the importance of a larger data set for the application of FDR estimation methods in order to guarantee reliable estimation of the proportion of true null hypotheses. The choice of control method mainly depends on the specific setting and the aims of an analysis. For example, when high specificity in testing of a limited number of hypotheses as in a replication study is desired, we recommend to utilize FWER methods rather than FDR methods.

## Additional files

Additional file 1 CKDGen study contributions assigned to discovery set for illustration of procedures controlling type I error. (DOCX 17 kb)

Additional file 2 CKDGen study contributions assigned to replication set for illustration of procedures controlling type I error. (DOCX 18 kb)

Additional file 3 Simulation – Average power and specificity for β_1_ = 1 and π_0_ = 75% (a), 50% (b), 25% (c). Applied procedures controlling the type I error: Bonferroni correction, Hommel’s procedure, Benjamini-Hochberg’s procedure, Two-stage procedure, Benjamini-Yekutieli’s procedure, Storey’s q-value method, Strimmer’s q-value method, Strimmer’s LFDR method. Power is defined as the proportion of correctly rejected hypotheses and specificity as the proportion of correctly maintained hypotheses. Both proportions potentially range from 0 to 1. Simulations for each scenario were repeated 100 times. (PNG 457 kb)

Additional file 4 Simulation – Observed estimations of π_0_ for Storey’s (qv) and Strimmer’s q-value methods (fdr)for β_1_ = 1 and π_0_ = 75% (a), 50% (b), 25% (c). (PNG 209 kb)

## Abbreviations

FDR: False discovery rate
FWER: Family-wise error rate
GWAS: Genome-wide association study
LD: Linkage disequilibrium
LFDR: Local false discovery rate
pFDR: Positive false discovery rate
SNP: Single nucleotide polymorphism

## References

[1] Balding DJ (2006). A tutorial on statistical methods for population association studies. Nat Rev Genet.

[2] Zeng P, Zhao Y, Qian C, Zhang L, Zhang R, Gou J, Liu J, Liu L, Chen F (2015). Statistical analysis for genome-wide association study. J Biomed Res.

[3] Rothman KJ, Greenland S, Lash TL (2008). Modern Epidemiology.

[4] Goeman JJ, Solari A (2014). Multiple hypothesis testing in genomics. Stat Med.

[5] Bland JM, Altman DG (1995). Multiple significance tests: the Bonferroni method. BMJ.

[6] Efron B (2001). Empirical Bayes analysis of a microarray experiment. J Am Stat Assoc.

[7] Storey JD (2002). A direct approach to false discovery rates. J R Stat Soc.

[8] Pattaro C, Teumer A, Gorski M, Chu AY, Li M, Mijatovic V, Garnaas M, Tin A, Sorice R, Li Y (2016). Genetic associations at 53 loci highlight cell types and biological pathways relevant for kidney function. Nat Commun.

[9] Benjamini Y, Hochberg Y (1995). Controlling the false discovery rate: a practical and powerful approach to multiple testing. J R Stat Soc.

[10] Holm SA (1979). Simple sequentially Rejective multiple test procedure. Scand J Stat.

[11] Li J, Ji L (2005). Adjusting multiple testing in multilocus analyses using the eigenvalues of a correlation matrix. Heredity.

[12] Pe'er I, Yelensky R, Altshuler D, Daly MJ (2008). Estimation of the multiple testing burden for genomewide association studies of nearly all common variants. Genet Epidemiol.

[13] Grams ME, Tin A, Rebholz CM, Shafi T, Kottgen A, Perrone RD, Sarnak MJ, Inker LA, Levey AS, Coresh J (2017). Metabolomic alterations associated with cause of CKD. Clin J Am Soc Nephrol.

[14] Hochberg Y (1988). A sharper Bonferroni procedure for multiple tests of significance. Biometrika.

[15] Hommel G (1988). A stagewise rejective multiple test procedure based on a modified Bonferroni test. Biometrika.

[16] Benjamini Y, Krieger AM, Yekutieli D (2006). Adaptive linear step-up procedures that control the false discovery rate. Biometrika.

[17] Benjamini Y, Yekutieli D (2001). The control of the false discovery rate in multiple testing under dependency. Ann Stat.

[18] Storey JD, Tibshirani R (2003). Statistical significance for genomewide studies. Proc Natl Acad Sci U S A.

[19] Strimmer K (2008). A unified approach to false discovery rate estimation. BMC Bioinform.

[20] The Comprehensive R Archive Network. https://cran.r-project.org/. Accessed 17 Oct 2017.

[21] Bioconductor. https://www.bioconductor.org/. Accessed 17 Oct 2017.

[22] Pollard KS, Dudoit S, van der Laan MJ. Multiple testing procedures: the multtest package and applications to genomics. In: Gentleman R., Carey VJ, Huber W, Irizarry RA, Dudoit S, editors. Bioinformatics and Computational Biology Solutions Using R and Bioconductor: Springer, New York; 2005.

[23] Storey JD, Bass AJ, Dabney A, Robinson D. qvalue: Q-value estimation for false discovery rate control. 2015. R package version 2.10.0. http://github.com/jdstorey/qvalue. Accessed 17 Oct 2017.

[24] MuToss Coding Team (Berlin 2010), Blanchard G, Dickhaus T, Hack N, Konietschke F, Rohmeyer K, Rosenblatt J, Scheer M, Werft W. mutoss: Unified Multiple Testing Procedures. 0.1–10 edn; 2015; The Mutoss package and accompanying mutossGUI package are designed to ease the application and comparison of multiple hypothesis testing procedures. https://CRAN.R-project.org/package=mutoss. Accessed 17 Oct 2017.

[25] Klaus B, Strimmer S. fdrtool: Estimation of (Local) False Discovery Rates and Higher Criticism. 2015: Estimates both tail area-based false discovery rates (Fdr) as well as local false discovery rates (fdr) for a variety of null models (p-values, z-scores, correlation coefficients, t-scores). The proportion of null values and the parameters of the null distribution are adaptively estimated from the data. In addition, the package contains functions for non-parametric density estimation (Grenander estimator), for monotone regression (isotonic regression and antitonic regression with weights), for computing the greatest convex minorant (GCM) and the least concave majorant (LCM), for the half-normal and correlation distributions, and for computing empirical higher criticism (HC) scores and the corresponding decision threshold. https://CRAN.R-project.org/package=fdrtool. Accessed 17 Oct 2017.

[26] CKDGen Consortium. Meta-Analysis Data. http://ckdgen.imbi.uni-freiburg.de/. Accessed 17 Oct 2017.

[27] Auton A, Brooks LD, Durbin RM, Garrison EP, Kang HM, Korbel JO, Marchini JL, McCarthy S, McVean GA, Abecasis GRA (2015). Global reference for human genetic variation. Nature.

[28] Purcell S, Neale B, Todd-Brown K, Thomas L, Ferreira MA, Bender D, Maller J, Sklar P, de Bakker PI, Daly MJ (2007). PLINK: a tool set for whole-genome association and population-based linkage analyses. Am J Hum Genet.

[29] Lai YA (2017). Statistical method for the conservative adjustment of false discovery rate (q-value). BMC Bioinformatics.

[30] Stephens M (2017). False discovery rates: a new deal. Biostatistics (Oxford, England).

