# Supplementary material for: Control procedures and estimators of the false discovery rate and their application in low-dimensional settings: an empirical investigation
Source: BMC Bioinformatics. 2018 Mar 2;19:78. doi: 10.1186/s12859-018-2081-x (PMC5833079; doi:10.1186/s12859-018-2081-x)
Supplement: Supplementary file 1 — CKDGen study contributions assigned to discovery set for illustration of procedures controlling type I error. (DOCX 17 kb) [file 12859_2018_2081_MOESM1_ESM.docx]

**Additional file 1: CKDGen study contributions assigned to discovery set for illustration of procedures controlling type I error**

| Study | Sample size | Study type | Reference for imputation | Category* |
| --- | --- | --- | --- | --- |
| 1. YFS | 2023 | population-based | 1000G imputed | 4 |
| 1. JUPITER | 8780 | RCT, healthy men/women with LDL<130 and CRP>=2 | 1000G imputed | 3 |
| 1. NESDA | 1856 | longitudinal, mainly cases with major depression, anxiety disorder | Hapmap imputed | 2 |
| 1. HPFS | 818 | nested case-control study, men only, all diabetics | Hapmap imputed | 2 |
| 1. HYPERGENES HTN cases | 1591 | nested case-control study, hypertensive patients | Hapmap imputed | 2 |
| 1. FHS | 7782 | prospective, families | Hapmap imputed | 1 |
| 1. CROATIA-KORCULA | 888 | cross-sectional, families | Hapmap imputed | 1 |
| 1. INGI-CILENTO | 821 | geographic restricted, cross-sectional, pedigree info | Hapmap imputed | 1 |
| 1. CROATIA-VIS | 768 | cross-sectional, families | Hapmap imputed | 1 |
| 1. GENOA | 1163 | families | Hapmap imputed | 1 |
| 1. ERF | 2561 | population-based, families | Hapmap imputed | 1 |
| 1. EGCUT 370K | 863 | population-based | Hapmap imputed | 1 |
| 1. WGHS | 21940 | prospective, women only | Hapmap imputed | 1 |
| 1. ORCADES | 704 | cross-sectional, families | Hapmap imputed | 1 |
| 1. RS-I | 4390 | prospective, population-based | Hapmap imputed | 1 |
| 1. DESIR | 715 | population-based | Hapmap imputed | 1 |
| 1. INGI-CARLANTINO | 447 | geographic restricted | Hapmap imputed | 1 |
| 1. AGES | 3219 | population-based | Hapmap imputed | 1 |
| 1. CROATIA-SPLIT | 478 | population-based | Hapmap imputed | 1 |
| 1. BLSA | 723 | population-based | Hapmap imputed | 1 |
| 1. AUSTWIN | 9592 | population-based, families | Hapmap imputed | 1 |
| 1. Amish | 1211 | ethnic-restricted, population-based | Hapmap imputed | 1 |
| 1. OGP-TALANA | 862 | geographic restricted | Hapmap imputed | 1 |
| 1. CHS | 2820 | prospective, exclusion of subjects with specific diseases | Hapmap imputed | 1 |
| 1. POPGEN | 1163 | prospective, population-based | Hapmap imputed | 1 |
| 1. INGI-VAL BORBERA | 1636 | geographic restricted, population-based, families | Hapmap imputed | 1 |
| 1. ASPS | 848 | prospective, exclusion of subjects with specific diseases | Hapmap imputed | 1 |
| 1. RS-II | 1863 | prospective, population-based | Hapmap imputed | 1 |
| 1. INGI-FVG | 874 | geographic restricted | Hapmap imputed | 1 |
| 1. NSPHS | 565 | cross-sectional, families | Hapmap imputed | 1 |
| 1. EGCUT Omni | 261 | population-based | Hapmap imputed | 1 |
| 1. MICROS | 1201 | geographic restricted, cross-sectional, population-based, extended pedigrees | Hapmap imputed | 1 |
| 1. HABC | 1663 | prospective | Hapmap imputed | 1 |
| 1. KORA-F4 | 1814 | prospective, population-based | Hapmap imputed | 1 |
| 1. HYPERGENES HTN ctrls | 1662 | nested case-control study | Hapmap imputed | 1 |
| total | 90.565 |  |  |  |

*Categories were defined in accordance to general study type (population-based vs diseased cohort) and imputation references (HapMap vs 1000Genomes). By conditioning on the presence of at least one study from each of the 4 derived categories in either settings and on a sample size ratio of 2:1, study contributions were randomly assigned to discovery set or replication set.
