# Supplementary material for: Control procedures and estimators of the false discovery rate and their application in low-dimensional settings: an empirical investigation
Source: BMC Bioinformatics. 2018 Mar 2;19:78. doi: 10.1186/s12859-018-2081-x (PMC5833079; doi:10.1186/s12859-018-2081-x)
Supplement: Supplementary file 2 — CKDGen study contributions assigned to replication set for illustration of procedures controlling type I error. (DOCX 18 kb) [file 12859_2018_2081_MOESM2_ESM.docx]

**Additional file 2: CKDGen study contributions assigned to replication set for illustration of procedures controlling type I error**

| Study | Sample size | Study type | Reference for imputation | Category* |
| --- | --- | --- | --- | --- |
| 1. 3C | 6431 | prospective, population-based | 1000G imputed | 4 |
| 1. Advance | 2287 | RCT, only diabetics | 1000G imputed | 3 |
| 1. PROSPER-PHASE | 5236 | RCT, subjects with vascular event or at high risk for vascular disease | Hapmap imputed | 2 |
| 1. NHS | 786 | nested case-control study, diabetics, women only | Hapmap imputed | 2 |
| 1. MESA | 2521 | community-based | Hapmap imputed | 1 |
| 1. SHIP | 3228 | prospective, population-based | Hapmap imputed | 1 |
| 1. KORA-F3 | 1641 | prospective, population-based | Hapmap imputed | 1 |
| 1. INCIPE | 940 | cross-sectional, population-based, age 40 years and older | Hapmap imputed | 1 |
| 1. FamHS | 3838 | families | Hapmap imputed | 1 |
| 1. HCS | 1235 | population-based | Hapmap imputed | 1 |
| 1. SORBS | 856 | geographic restricted, population-based | Hapmap imputed | 1 |
| 1. SHIP-TREND | 986 | prospective, population-based | Hapmap imputed | 1 |
| 1. BMES | 2437 | prospective | Hapmap imputed | 1 |
| 1. ARIC | 8982 | prospective, population-based | Hapmap imputed | 1 |
| 1. SAPALDIA | 1444 | population-based | Hapmap imputed | 1 |
| total | 42.848 |  |  |  |

*Categories were defined in accordance to general study type (population-based vs diseased cohort) and imputation references (HapMap vs 1000Genomes). By conditioning on the presence of at least one study from each of the 4 derived categories in either settings and on a sample size ratio of 2:1, study contributions were randomly assigned to discovery set or replication set.
