# Supplementary figures and images for: Control procedures and estimators of the false discovery rate and their application in low-dimensional settings: an empirical investigation
Source: BMC Bioinformatics. 2018 Mar 2;19:78. doi: 10.1186/s12859-018-2081-x (PMC5833079; doi:10.1186/s12859-018-2081-x)

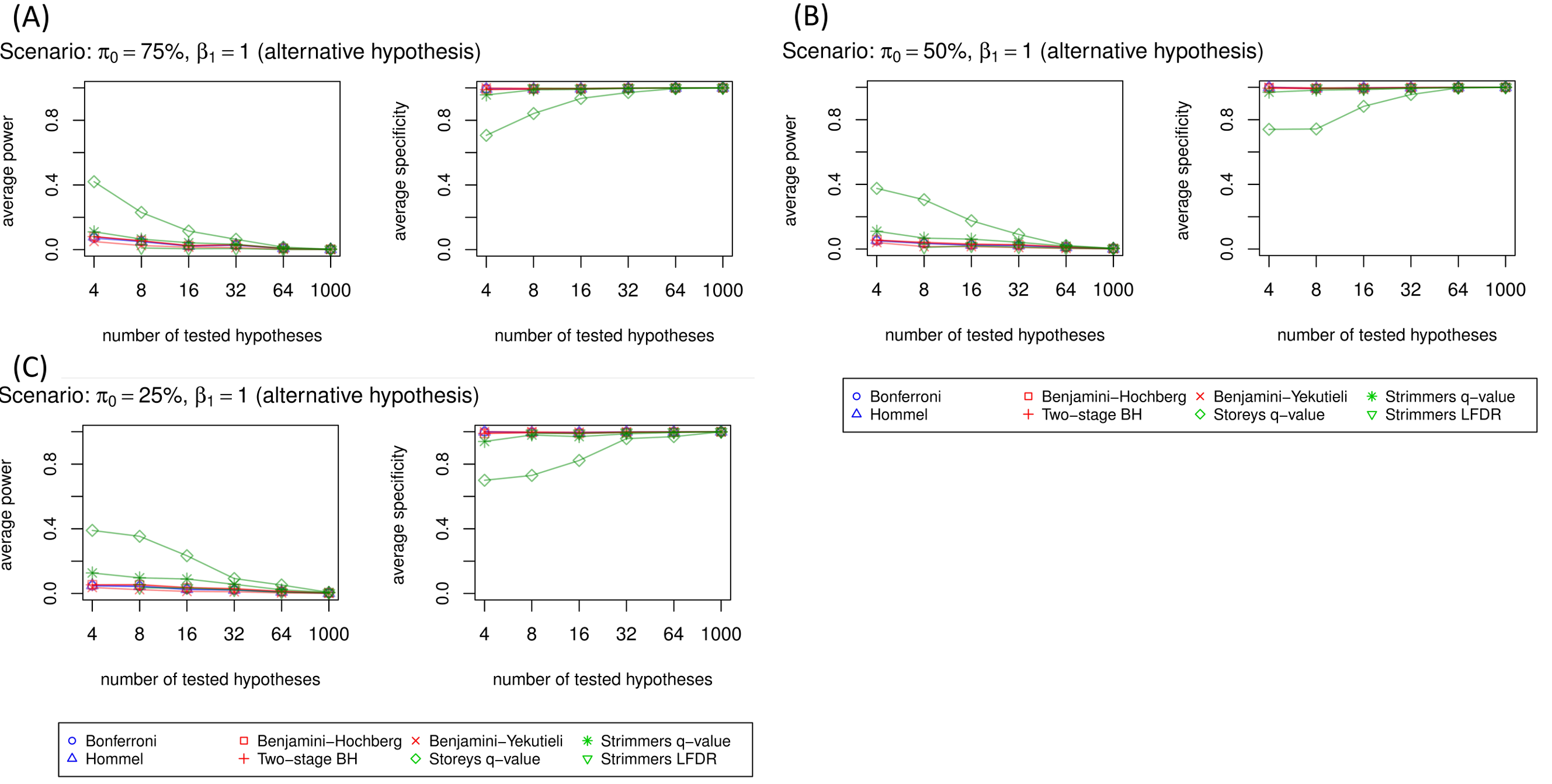

Supplement: Supplementary file 3 — Simulation – Average power and specificity for β1 = 1 and π0 = 75% (a), 50% (b), 25% (c). Applied procedures controlling the type I error: Bonferroni correction, Hommel’s procedure, Benjamini-Hochberg’s procedure, Two-stage procedure, Benjamini-Yekutieli’s procedure, Storey’s q-value method, Strimmer’s q-value method, Strimmer’s LFDR method. Power is defined as the proportion of correctly rejected hypotheses and specificity as the proportion of correctly maintained hypotheses. Both proportions potentially range from 0 to 1. Simulations for each scenario were repeated 100 times. (PNG 457 kb) [file 12859_2018_2081_MOESM3_ESM.png]

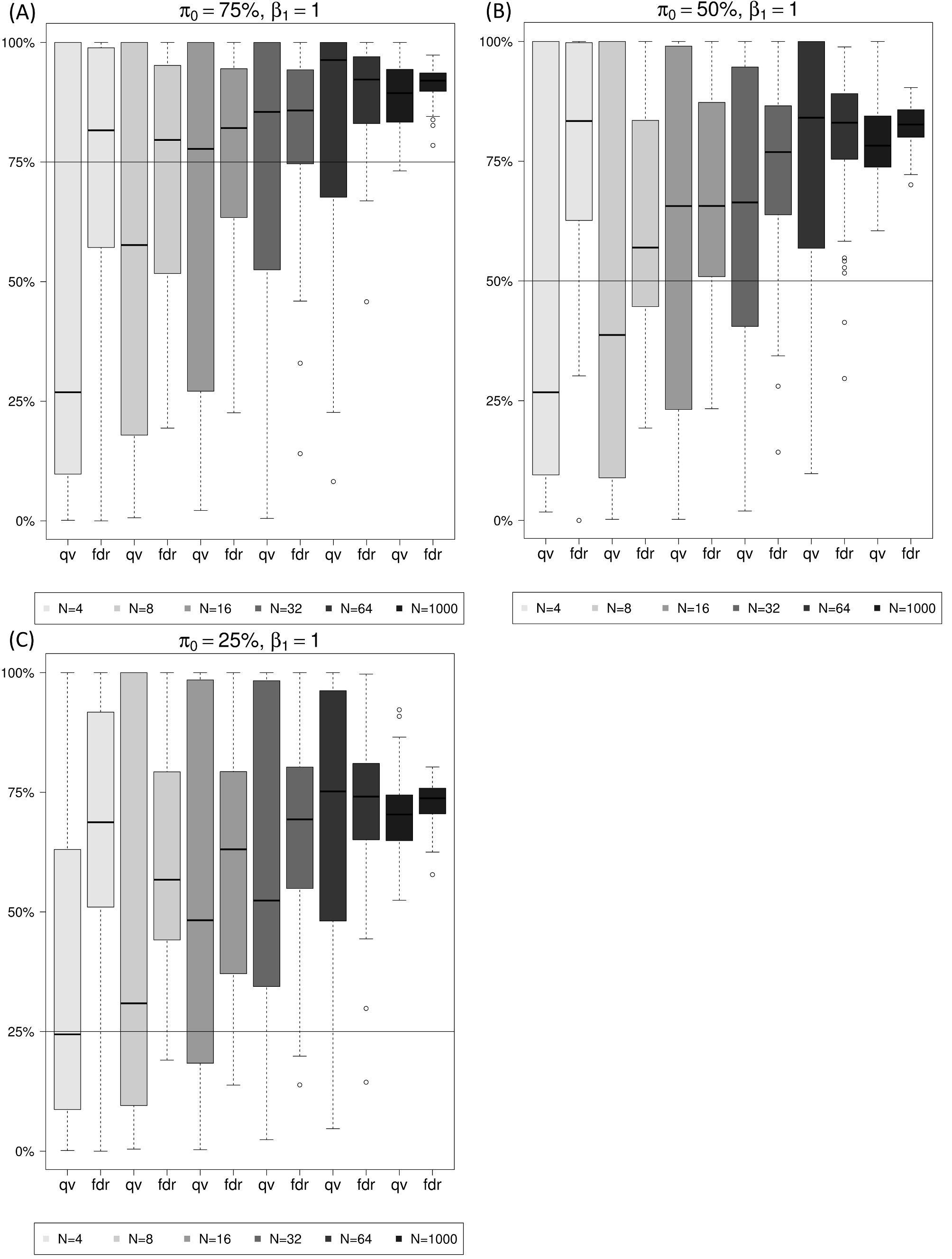

Supplement: Supplementary file 4 — Simulation – Observed estimations of π0 for Storey’s (qv) and Strimmer’s q-value methods (fdr)for β1 = 1 and π0 = 75% (a), 50% (b), 25% (c). (PNG 209 kb) [file 12859_2018_2081_MOESM4_ESM.png]
